# Demonstration videos of psychodynamic and systemic techniques in clinical psychology education

**DOI:** 10.1038/s41598-026-51978-x

**Published:** 2026-05-06

**Authors:** Christin Hannse Reinhardt, Christoph Kröger

**Affiliations:** Department of Clinical Psychology and Psychotherapy, University of Hildesheim, Hildesheim, Germany

**Keywords:** Demonstration video, Clinical skills, Modelling, Psychotherapy training, University teaching, Standardised simulation, Health care, Psychology, Psychology

## Abstract

Demonstration videos are an innovative method for teaching approach-specific techniques. This randomised-controlled study examines the impact of approach-specific demonstration videos on students’ practical skills. Undergraduate psychology students (*N* = 123) received approach-specific instructional texts and were then randomised into one of four groups in preparation for a standardised simulation. In two intervention groups (IGs), students watched a video on either psychodynamic or systemic techniques, whereas the control groups (CGs) reviewed the respective instructional text again. In the real-life simulation with a standardised patient, trained psychologists rated approach-specific techniques using behavioural anchors. Students in the systemic IG performed significantly better in systemic techniques (*M* = 3.91) than those in the systemic CG (*M* = 3.11), *p* < .001, *d* = 1.03. There was no significant difference in the implementation of psychodynamic techniques between the psychodynamic IG (*M* = 3.22) and CG (*M* = 3.41), *p* = .477, *d* = − 0.19. These findings indicate that students can practically implement techniques from demonstration videos. Videos requiring a more comprehensive theoretical background and insight into psychotherapists’ thought processes might be less beneficial for undergraduate students. Further studies are necessary to explore the long-term development of skills through demonstration videos.

**Supplementary Information:**

The online version contains supplementary material available at 10.1038/s41598-026-51978-x.

Therapeutic skills and abilities should be acquired early on in psychology studies to lay the groundwork for competent clinical practice^1^. Over the past few decades, competency-based learning has become more prominent^2–5^. Evaluating the impact of innovative teaching methods on clinical competencies is essential for ensuring a high-quality clinical psychology education.

Demonstration videos, as an innovative teaching method, present sequences from therapeutic interactions between a model therapist and a standardised patient (SP)^6^. Complementing the knowledge gained through lectures and seminars, demonstration videos can prepare students for practical examinations and patient contact during internships. Model therapists in demonstration videos can present both generic competencies (e.g., building a therapeutic alliance) and approach-specific competencies (e.g., Socratic questioning)^1,7^.

According to the Observational Learning Theory^8^, students should be able to reproduce behaviours observed in a demonstration video. Research suggests that especially beginner students often seek therapeutic models to imitate in order to quickly develop clinical skills^9^. Various theoretical models describe stages of skill development^10–12^. Miller’s Hierarchical Model of Clinical Competencies^13^ defines four developmental stages: (1) Knowledge (*knows*), (2) Application (*knows how*), (3) Performance (*shows how*), (4) Action in Practice (*does*). By observing a model therapist performing a specific technique in a video, beginner students may acquire the practical knowledge (stage 2) necessary to implement the technique themselves in a standardised simulation (stage 3).

Early skill acquisition could be particularly important for undergraduate students, who are often referred to as novices^14^. Considering the five-stage model of therapeutic competence^15^, which includes the stages of novice, advanced beginner, competence, proficiency, and expertise, quicker development from stage to stage could lead to achieving expertise sooner. Earlier expertise can positively influence patients’ outcomes, since therapist effects influence the treatment success^16^.

In medical education, demonstration videos have long been successfully deployed to prime students for Objective Structured Clinical Examinations and the training of clinical competencies^17–19^. Although demonstration videos are widely used in psychology education, there is limited evaluation research. Evidence suggests that videos focused on a particular therapeutic approach can enhance psychology students’ related declarative knowledge^6^. In a randomised-controlled trial (RCT) on students’ implementation of cognitive behavioural skills^20^, the intervention group (IG; demonstration video + manual) outperformed the control group (CG; manual). This suggests that, besides increasing declarative knowledge, demonstration videos may enhance the practical application of psychotherapeutic skills. However, as different psychotherapy approaches require specific skills^1^, it remains unclear whether videos presenting other therapeutic approaches can likewise improve practical approach-specific skills.

This RCT examines whether additional demonstration videos presenting psychodynamic or systemic techniques can improve practical approach-specific skills of undergraduate psychology students. We hypothesise that in a standardised simulation, students in the IGs (demonstration video + instructional text) would apply the required approach-specific techniques better than those in the CGs (instructional text).

## Methods

The participants in this study were undergraduate psychology students (*N* = 123) enrolled in the five Basic Conversational Skills courses at the University of Hildesheim during the winter semester 2023/2024 (female: *n* = 96 [78%]; 5th Semester: *n* = 110 [89.4%]). In previous semesters, students had attended a lecture and at least one seminar on mental disorders. The study was approved by the Ethics Committee of the University of Hildesheim (Code 292). All research procedures were conducted in full accordance with the relevant guidelines and regulations, including the Declaration of Helsinki. Prior to participation, each student provided written informed consent. All students had to complete a practical real-life simulation as a mandatory course requirement. After participation, the students received all learning materials used in the study.

## Study design & procedure

Our study design investigated preparation materials for psychodynamic techniques (IG 1 vs. CG 1) and for systemic techniques (IG 2 vs. CG 2). Hence, we present two studies in one. The results of the two studies cannot be directly compared due to differences in the preparation materials and the approach-specific outcome measures.

**Fig. 1:**
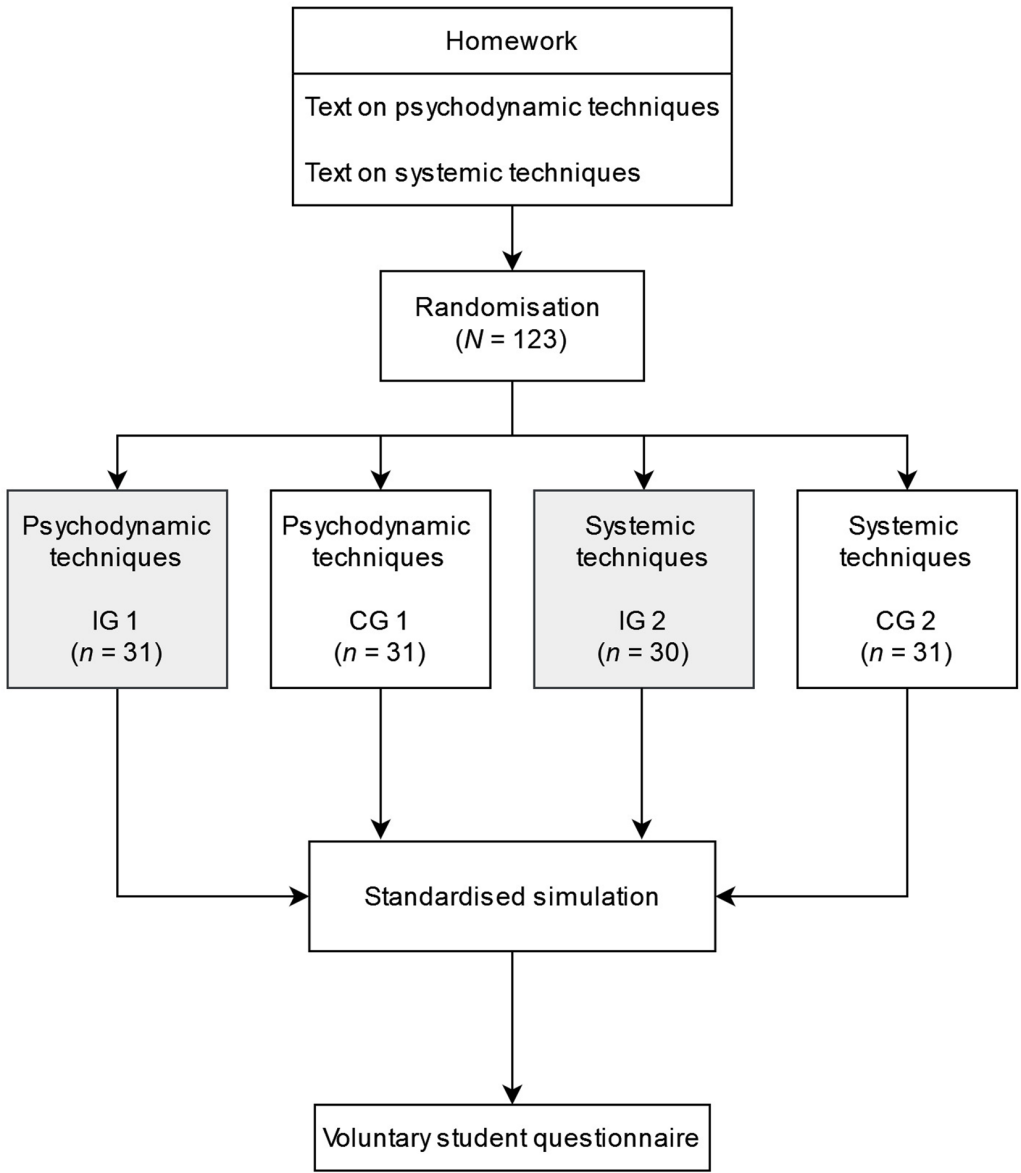
Study Procedure. The procedure of the randomised-controlled trial is illustrated. Students were randomly assigned to one of four groups: Intervention group 1 (IG 1), control group 1 (CG 1), intervention group 2 (IG 2), and control group 2 (CG 2).

Figure 1 shows the study procedure. To gain approach-specific knowledge at semester start, all students read an instructional systemic text (solution-focused techniques) and an instructional psychodynamic text (clarifying, confronting, and interpreting) as homework. All participating students reported that they had read the assigned homework texts in preparation for the simulation task. Before the standardised simulation, the students were randomly assigned to one of four groups (see Fig. 1). The IGs watched either a video on psychodynamic or systemic techniques, while the CGs reviewed the approach-specific homework texts again. In the 14-minute real-life simulation with a standardised patient (SP), two independent raters evaluated the students’ application of approach-specific techniques using behavioural anchors. After the simulation, the raters provided individual feedback to the students. Finally, the students could voluntarily fill out a student questionnaire that measured the students’ theoretical knowledge, practical experience, interest in psychotherapy, empathy, and their psychological mindedness.

### Preparation materials

#### Video & text on psychodynamic techniques (IG 1 & CG 1)

In the video, a licensed therapist demonstrated the psychodynamic techniques of *clarifying*,*confronting*,* and interpreting* based on the case example of an SP who indicated a guilt conflict. The video focused on the model therapist’s behaviour, while the symptoms displayed by the SP were described only briefly to provide enough context for understanding the demonstrated techniques. As cues might help students to transfer their knowledge and integrate information^21^, we displayed the interpretation types (e.g., content interpretations, interpreting defence mechanisms) as subtitles in the video. The instructional homework text on *clarifying*,* confronting*,* and interpreting* was an extract from a textbook^22^. Example formulations outlined different interpretation forms. The video was 12 minutes long and the text (also made available for 12 minutes) had 766 words.

## Video & text on systemic techniques (IG 2 & CG 2)

In the video, another licensed therapist demonstrated the *miracle question* from Solution-Focused Brief Therapy (SFBT)^23^ and subsequently explored the fictional day with the SP through *related questions* in the present tense to create a solution trance (e.g., how does your day continue? How does it feel? ). The video focused on the model therapist. The depressive symptoms of the SP were indicated subtly. The instructional homework text regarding SFBT was an extract from a textbook^24^. It contained an example formulation of *the miracle question and related questions* to explore the fictional miracle day. Both the video on SFBT techniques (six minutes) and the text with 601 words were provided to the students for six minutes.

## Standardised patients, raters & simulation task

The SPs were trained graduate psychology students (*N* = 3; all female). The *Supplemental Case Vignettes S1* contain summarised and translated scripts. The role scripts comprehensively describe a patient with bulimia nervosa, characterised by an individuation versus dependency conflict (IG 1 & CG 1), and a patient with social anxiety disorder (IG 2 & CG 2).

Four trained psychologists (*N* = 4) served as raters. For each simulation, two of them simultaneously observed and independently rated the students’ implementation of the approach-specific techniques. All raters had prior experience in evaluating students in simulations and were blind to the students’ group affiliation.

The 14-minute simulation consisted of a six-minute basic conversational skills segment, a five-minute approach-specific segment (either psychodynamic techniques or systemic techniques), a two-minute segment dealing with therapy resistance, and a one-minute segment on managing emotions. Depending on the group assignment, the students had to apply the psychodynamic techniques, *clarify*,* confront*,* and interpret* or apply the *miracle question* from the systemic therapy approach, along *with related questions*, to create a solution trance (“how do you feel?“, “What do you do?“, and “How does someone else notice that the miracle has happened?“). The students had to implement the learned techniques on a new case example. Therefore, the simulation was a transfer task^25,26^. The students had to continuously adjust the techniques in response to the SPs’ reactions, drawing on both approach-specific and disorder-specific knowledge from lectures and seminars. The simulation task aimed to connect students’ knowledge of psychological disorders or psychodynamic conflicts with the approach-specific techniques from the preparation materials.

## Measurements

### Approach-specific techniques

Based on two approach-specific books^27,28^ we developed behavioural anchors to examine the practical implementation of the approach-specific techniques, since no suitable, valid, and reliable measurement instrument existed. For the participants in the psychodynamic groups, we assessed the use of *clarifications*,* confrontations*,* and interpretations* using ten specific items. For the participants in the systemic groups, another ten items were used to measure the application of systemic techniques, including the *miracle question and related questions*. All approach-specific items were measured on a seven-point Likert scale from 1=* strongly disagree* to 7 = *strongly agree* (see *Supplemental Ratings S2*). A psychodynamic item, for example, was: *The student expressed contradictions in the statements or between the statements and/ or nonverbal signals of the SP*. One example of a systemic item was: *The student prevented the SP from slipping into a problem-focused perspective and ensured that the SP remained focused on a solution-oriented viewpoint.* For each student, we computed the mean rating across the 10 items corresponding to their respective approach (*systemic* or *psychodynamic*), separately for both raters. To determine the final scores, we averaged over the two ratings. The internal consistency values were very good and good (*psychodynamic techniques*: Cronbach’s α = 0.86; *systemic techniques*: Cronbach’s α = 0.76). The intraclass correlation coefficients (one-way random effects, single measure) indicated good reliability^29,30^ for both the *psychodynamic* (ICC = 0.80) and the *systemic techniques* (ICC = 0.83).

## Student questionnaire

The Cronbach’s alpha values ranged from acceptable to good for the collected variables. The values are listed in the *Supplemental Table 1S3*. The means and standard deviations for all collected variables are in the *Supplemental Table 2S4*.

## Theoretical knowledge

Eight multiple-choice questions tested the general knowledge and understanding gained from approach-specific lectures and the preparation materials. They contained the core skills in therapy - open questions, paraphrasing, validation - as well as two questions regarding the SFBT and the miracle question, and two questions about clarifying, confronting, and interpreting (e.g., *What considerations are important when presenting the miracle question and related questions to a patient?*). We used the guess-rate correction in evaluating the questions. Students could achieve one point, half a point (more than 50% answered correctly), or zero points. We then computed the total score.

### Practical experience

Five items measured the practical clinical experience on a five-point Likert scale from *does not apply at all* (1) to *fully applies* (5). The students were asked about internships, job shadowing, counselling experience, and whether they practised therapeutic skills in role plays (e.g., *I am currently working as a consultant.*). A total value was computed.

### Interest in psychotherapy

Using four items, we rated interest in the profession of psychotherapists, in taking the licensing examination, in different psychotherapy approaches, and in systemic therapy or psychodynamic therapy, specifically, on a six-point Likert scale from *strongly disagree* (0) to *strongly agree* (5). The fourth item differed depending on the group affiliation (IG and CG 1: *I am interested in psychodynamic therapy.*; IG and CG 2: *I am interested in systemic therapy.*). Therefore, we computed the overall score twice.

### Interpersonality Reactivity Index (IRI)

Empathy was measured using the German version of the *Saarbrücken Personality Questionnaire*^31^. The original version is the *IRI*^32,33^. The subscales *Perspective Taking* (PT), *Fantasy* (FS), *Empathic Concern* (EC), and *Personal Distress* (PD) measure different empathy aspects with four items each (original version: Cronbach’s α = 0.71–0.78). For example, one PT item states: *Before criticising somebody*,* I try to imagine how I would feel if I were in their place*. The accuracy of the 16 statements was self-assessed using a five-point Likert scale from *never* (1) to *always* (5).

### Balanced Index of Psychological Mindedness (BIPM)

We used the German version^34^ of the BIPM^35^ with 14 Items and two subscales (German version *BIPM total*: Cronbach’s α = 0.80, *interest*: Cronbach’s α = 0.75; *insight*: Cronbach’s α = 0.75). The self-assessment questionnaire determined psychological mindedness using a five-point Likert scale varying from *does not apply at all* (0) to *fully applies* (4). A sample item is: *I am better off when being in touch with my feelings.* We computed the mean between the subscales.

### Data analysis

We analysed the data using SPSS Statistics (Version 29.0). Descriptive statistics were calculated for all collected variables. After checking the statistical assumptions, we conducted independent samples *t*-tests to investigate group differences in the implementation of approach-specific techniques. Due to multiple testing, we applied a Bonferroni correction and adjusted the significance level to α = 0.025 for each of the two primary *t*-tests^36^. Group differences regarding potential confounding variables were examined using a Mann-Whitney *U* test and *t*-tests. To control for confounding variables, group differences among the systemic groups were further assessed using analysis of covariance. As indicated by a post-hoc power analysis conducted with G*Power, the statistical power of the independent primary *t*-tests was low (0.14). Thus, the *t*-tests were insufficient to reliably detect small effect sizes of *d* = 0.18 due to the small sample size.

## Results

### Participants

All students completed the simulation (*N* = 123). A total of 111 students filled out the voluntary student questionnaire. Three of these students did not complete the questionnaire or left parts of it blank. Table 1 presents the descriptive statistics for the four groups. Descriptive statistics for the investigated covariates are depicted in *the Supplemental Table 2S4*.

**Table 1:** Descriptive Statistics. The percentage distribution of male and female students, as well as the number of semesters studied, are presented for each group (IG: Intervention group, CG: Control group). Additionally, for each group, the mean and standard deviation regarding the implementation of approach-specific techniques are reported. Significant differences (*p* < .001) between the groups implementing the systemic techniques are marked with asterisks.

	Psychodynamic techniques			Systemic techniques			
	IG 1	CG 1		IG 2	CG 2		Total score
Gender: Female *n* (%) Male *n* (%)	24 (77.4) 7 (22.6)	24 (77.4) 7 (22.6)		27 (90) 3 (10)	21(67.7) 10 (32.3)		96 (78) 27 (22)
Number of Semesters: 5 *n* (%) 7 *n* (%) 13 *n* (%)	29 (93.5) 2 (6.5) -	26 (83.9) 4 (12.9) 1 (3.2)		27 (90) 3 (10) -	28 (90.3) 3 (9.7) -		110 (89.4) 12 (9.8) 1 (0.8)
Approach-Specific Techniques: *M* (*SD*)	*n* = 31 3.22 (1.03)	*n* = 31 3.41 (1.08)		*n* = 30 3.91 (0.72) ***	*n* = 31 3.11 (0.84) ***		*N* = 123 3.41 (0.97)

### Psychodynamic techniques

**Fig. 2:**
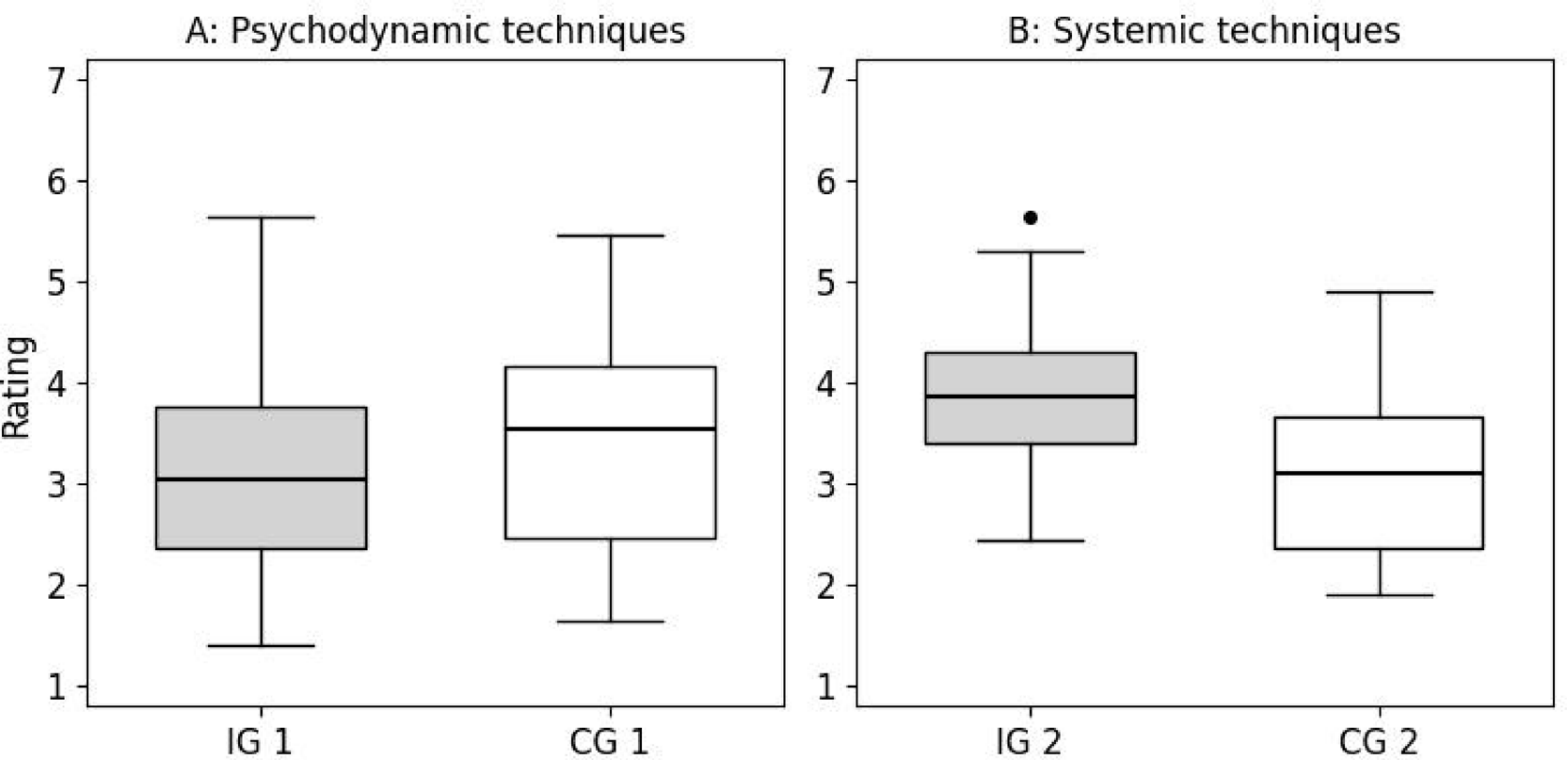
Group Differences in Practical Implementation. Panel A shows group differences between intervention group 1 (IG 1) and control group 1 (CG 1) in the implementation of the psychodynamic techniques. Panel B depicts the differences between intervention group 2 (IG 2) and control group 2 (CG 2) in the implementation of systemic techniques. The implementation of both approach-specific techniques was rated on a Likert scale from 1 to 7.

We did not find a group difference between the groups IG1 and CG 1, which implemented the psychodynamic techniques of *clarification*,* confrontation*,* and interpretation*, *t* (60) = − 0.72, *p* = .477, *d* = − 0.19 (see Fig. 2A). Furthermore, we did not observe group differences between the two groups regarding the confounding variables.

### Systemic techniques

The IG 2 (*n* = 30, *M* = 3.91, *SD* = 0.72) implemented the *miracle question and the related questions* from the SFBT better than the CG 2 (*n* = 31, *M* = 3.11, *SD* = 0.84), *t* (59) = 4.02, *p* < .001, *d* = 1.03 (see Fig. 2B*)*.

### Group differences in practical implementation

The two groups implementing the systemic techniques differed in their self-reported empathic concern (IG 2: *n* = 25, *Mdn* = 18.00; CG 2: *n* = 29, *Mdn* = 15.00), *U* = 225.50, *Z* = − 2.40, *p* < .05, *r* = − .33, and their self-reported personal distress, *t* (53) = 2.63, *p* < .05, *r* = .34 (see *supplemental Table 2S4)*. After adjusting for theoretical knowledge, practical experience, interest in psychotherapy, the empathy subscales, and psychological mindedness, the practical performance still differed between the groups that implemented the systemic techniques (IG 2: *n* = 22, CG 2: *n* = 28), *F* (1, 40) = 9.62, *p* < .05, η² = 0.19.

## Discussion

This RCT investigated the effect of approach-specific demonstration videos on students’ practical skills in standardised simulations. Students who watched an additional demonstration video on systemic techniques implemented these techniques better in their simulations with the SP than the CG, who repeated the instructional text. This suggests a benefit of the demonstration video for the practical implementation of systemic techniques. This finding is consistent with a previous study on a video presenting cognitive behavioural techniques^20^. Compared to the instructional text, the video on systemic techniques may have helped the students to gain a better understanding of the timing of questions and both non-verbal and verbal behaviour during the intervention. Thus, learning with the demonstration video may have enhanced students’ *know how*^13^, which in turn may have translated into a superior *show how* performance compared to the CG.

In contrast to our hypothesis, we did not find a difference between the IG and CG in implementing the psychodynamic techniques. With an additional demonstration video, the IG performed the techniques of clarification, confrontation, and interpretation comparably to the CG, who repeated an instructional text on psychodynamic techniques. Theoretical knowledge is an essential step towards the skilled implementation of techniques and for improving clinical competencies (first competency level, *know how*)^13^. Therefore, we theorise that undergraduate students may need more theoretical background knowledge to fully understand and model the demonstration video on the psychodynamic techniques, such as the conflict levels according to Operationalised Psychodynamic Diagnosis^37^, and a detailed book on the techniques clarification, confrontation, and interpretation^38^.

Additionally, the psychodynamic techniques learned may require a high degree of adaptability to the individual case example in the simulation, which may be challenging for undergraduate students. Given the rather limited time in the simulation compared to a 50-minute psychotherapy session, the students may not have been able to develop their own case conceptualisation focusing on intrapsychic conflicts and suppressed emotions. Therefore, it may have been more challenging for the students to formulate interpretations^39^. Also, identifying unconscious elements may be difficult to observe in a demonstration video that does not make the cognitive processes of the model therapist between their verbal interventions explicit. Nonetheless, a previous finding suggests that competency-oriented training formats may also be significant for psychodynamic techniques^40^. For psychodynamic approaches, it may therefore be helpful to use explanatory videos or book chapters that elaborate on the therapist’s thought processes.

### Limitations and future research

We used data from only one cohort at one university, resulting in a small sample size and limited statistical power. We measured the confounding variables after the simulation for organisational reasons. Ideally, the confounding variables would also have been measured both before and after the video or text. We tested only one video per approach, which limited the generalizability of our results. While the study aimed at knowledge transfer, it cannot be ruled out that students partially mimicked the videos. The behavioural anchors should be further evaluated on an independent sample. Since the study was conducted with undergraduate students, we used SPs and were therefore unable to investigate the effect of learned practical skills on actual patient outcomes.

Future studies should evaluate a wider range of demonstration videos, supplemented with additional explanatory videos to enhance understanding. Furthermore, future research should not only use textual cues but also visual cues in videos to highlight aspects of the SP’s behaviour (e.g., nonverbal reactions) that are most relevant for the learned techniques^21^. Learned practical skills could be assessed in graduate students who apply approach-specific techniques under supervision with real patients. Furthermore, follow-up studies should compare different presentations of the same approach-specific techniques to investigate the effects of therapists’ characteristics in videos. The long-term skill development through demonstration videos should be investigated using larger samples by including different universities.

## Conclusion

Demonstration videos provide an innovative teaching method that can contribute to students’ acquisition of practical psychotherapeutic skills. When using demonstration videos in teaching, students must be sufficiently familiar with the theoretical backgrounds of the demonstrated technique. Some demonstration videos may require additional explanatory materials to further support skill acquisition.

## Supplementary Information

Below is the link to the electronic supplementary material.

Supplementary Material 1

Supplementary Material 2

## Data Availability

For open data see Supplements.

## Open science statements

For materials (ratings and case vignettes) see Supplements. Transcribed videos can be provided by the authors upon request.
