# Supplementary material for: Demonstration videos of psychodynamic and systemic techniques in clinical psychology education
Source: Sci Rep. 2026 May 6;16:14390. doi: 10.1038/s41598-026-51978-x (PMC13149835; doi:10.1038/s41598-026-51978-x)
Supplement: Supplementary file 2 — Supplementary Material 2 [file 41598_2026_51978_MOESM2_ESM.docx]

# **Supplemental Case Vignettes S1**

##### **Case vignette 1 – Implementing Psychodynamic Techniques**

# *The 27-year-old female patient, diagnosed with bulimia nervosa, recounts developing an eating disorder during her youth. As a child, she struggled with being overweight. Furthermore, her mother's derogatory comments about her body affected her self-esteem. The patient had a distant relationship with her parents, as they were often busy working and had little time for family meals. Consequently, food for the patient was pre-prepared and portioned out. Sweets were readily available at home, although the patient got into trouble for eating them. After a breakup with her first boyfriend at fifteen, the patient's heartache led to a drastic decrease in her food intake. Any food she consumed resulted in vomiting due to emotional distress. The patient began to enjoy losing weight and adopted binge-eating followed by purging as a coping mechanism. Currently employed in a company, she is on the brink of a promotion to project manager, intensifying her anxiety about meeting the job's demands. During visits to her parents' home, the urge to purge escalates, exacerbated by the unhealthy, calorie-rich meals her mother prepares. Although she craves independence in choosing her food, she feels unable to assert her preferences in her parent's household. The desire to have control over her meals with her family remains unfulfilled.*

#####

##### **Case Vignette 2 – Implementing Systemic Techniques**

# *A 32-year-old female patient diagnosed with social anxiety disorder lives together with her fiancé. The patient completed her studies in German and History education and is currently doing her internship as a teacher. She is under significant pressure due to classroom observations and fears of failing. Since her school days, the patient has been afraid of evaluative situations, such as giving presentations. In elementary school, she was humiliated by her math teacher in front of the class when she couldn't solve a problem. This incident triggered her extreme anxiety about performance situations; she fears that she will blush, say something wrong, and be judged negatively by others. As a safety behaviour in the mornings, the patient wears a lot of makeup, avoids wearing white clothes, and meticulously plans what to say before class. The pressure she places on herself causes her to frequently stay up late at night, meticulously preparing her lessons. In her personal life, the patient has limited social contacts outside of two close friends and her fiancé. She avoids parties. Her fiancé wants to take a dance class with her, but her anxiety about embarrassing herself in front of others is too overwhelming. During one classroom observation, the patient faced a moment where she could not recall an answer to a student's question about a historical event. This unexpected situation caused her severe anxiety; she began to stutter, her face turned bright red, and she felt an overwhelming sense of panic. The examiner's facial expression seemed to confirm her worst fears—that she was making a fool of herself. Later, this incident caused her significant distress and reinforced her belief that she might not be fit for the teaching profession. As a result, she has since been doubling her efforts in lesson preparations, driven by the fear of experiencing a similar event in future observations.*

#

# **Supplemental Ratings S2**

**Ratings – Psychodynamic Techniques**

|  | Strongly disagree | | |  | Strongly agree | | |
| --- | --- | --- | --- | --- | --- | --- | --- |
| Clarification, Confrontation, and Interpretation | **1** | **2** | **3** | **4** | **5** | **6** | **7** |
| The student |  |  |  |  |  |  |  |
| 1. … clarified (e.g., restated, reorganized) the available SP’s information and tried to expand on information (e.g., asking further questions). | 1 | 2 | 3 | 4 | 5 | 6 | 7 |
| 1. … used questions and statements that logically followed the statements of the SP. | 1 | 2 | 3 | 4 | 5 | 6 | 7 |
| 1. … used purposeful questions and statements to gain an understanding of the SP’s behaviour/ thoughts/ feelings. | 1 | 2 | 3 | 4 | 5 | 6 | 7 |
| 1. … guided the SP towards potentially repressed emotions to facilitate insight for interpretation. | 1 | 2 | 3 | 4 | 5 | 6 | 7 |
| 1. … incorporated the SP’s nonverbal behaviour (e.g., during confrontation). | 1 | 2 | 3 | 4 | 5 | 6 | 7 |
| 1. … expressed contradictions in the statements or between the statements and/ or nonverbal signals of the SP. | 1 | 2 | 3 | 4 | 5 | 6 | 7 |
| 1. … confronted the SP with an uncomfortable aspect or inconsistencies in what was said to bring it to awareness and further deepen the processing. | 1 | 2 | 3 | 4 | 5 | 6 | 7 |
| 1. … presented a hypothesis-driven, comprehensible interpretation. | 1 | 2 | 3 | 4 | 5 | 6 | 7 |
| 1. … connected a behaviour/ feeling/ thought of the SP from the present with unconscious material in the interpretation. | 1 | 2 | 3 | 4 | 5 | 6 | 7 |
| 1. … included a relevant aspect from the SP’s present or past in the presented interpretation. | 1 | 2 | 3 | 4 | 5 | 6 | 7 |

*Note.* SP: Standardised patient. The items were translated.

|  | Strongly disagree | | |  | Strongly agree | | |
| --- | --- | --- | --- | --- | --- | --- | --- |
| Miracle question and related questions (Solution-Focused Brief Therapy) | **1** | **2** | **3** | **4** | **5** | **6** | **7** |
| The student |  |  |  |  |  |  |  |
| 1. … asked the miracle question at an appropriate pace, with short pauses, and comprehensible. | 1 | 2 | 3 | 4 | 5 | 6 | 7 |
| 1. … encouraged the SP to use positive phrasing through targeted questions (e.g., What did you do or feel instead?). | 1 | 2 | 3 | 4 | 5 | 6 | 7 |
| 1. … independently anticipated positive phrasing. | 1 | 2 | 3 | 4 | 5 | 6 | 7 |
| 1. … asked about different perspectives. | 1 | 2 | 3 | 4 | 5 | 6 | 7 |
| 1. … asked about specific behaviours through which the miracle would be noticed. | 1 | 2 | 3 | 4 | 5 | 6 | 7 |
| 1. … asked about feelings through which the miracle would be noticed. | 1 | 2 | 3 | 4 | 5 | 6 | 7 |
| 1. … explored various aspects of daily life. | 1 | 2 | 3 | 4 | 5 | 6 | 7 |
| 1. … probed deeper into at least one area to induce a brief solution-focused trance. | 1 | 2 | 3 | 4 | 5 | 6 | 7 |
| 1. … focused on the symptoms/complaints of the current problem and/ or how to manage them. | 1 | 2 | 3 | 4 | 5 | 6 | 7 |
| 1. … prevented the SP from slipping into a problem-focused perspective and ensured that the SP remained focused on a solution-oriented viewpoint. | 1 | 2 | 3 | 4 | 5 | 6 | 7 |

**Ratings – Systemic techniques**

*Note.* SP: Standardised patient. Items three and nine are inverted. The items were translated.

**Supplemental Table 1 S3**

| **Student questionnaire** | **Cronbach**’**s alpha** |
| --- | --- |
| Theoretical knowledge | α = .67 |
| Practical experience | α = .67 |
| Interest in …  systemic therapy  psychodynamic therapy | α = .86  α = .80 |
| IRI:  *Empathic Concern*  *Perspective Taking*  *Fantasy*  *Personal distress* | α = .74  α = .67  α = .66  α = .70 |
| BIMP:  *Insight*  *Interest* | α = .78  α = .83 |

*Internal Consistency of the Student Questionnaire*

*Note.* IRI: Interpersonality Reactivity Index; BIMP: Balanced Index of Psychological Mindedness.

**Supplemental Table 2 S4**

*Means and Standard deviations of Confounding variables*

|  | Psychodynamic groups | |  | Systemic groups | |  |  |
| --- | --- | --- | --- | --- | --- | --- | --- |
|  | IG 1 | CG 1 |  | IG 2 | CG 2 |  | Total score |
| Theoretical Knowledge:  *M* (*SD*) | *n* = 28  4.23 (1.38) | *n* = 26  4.65 (1.37) |  | *n* = 25  4.68 (1.03) | *n* = 30  4.90 (0.93) |  | *N* = 109  4.62 (1.20) |
| Practical Experience:  *M* (*SD*) | *n* = 30  10.07 (4.50) | *n* = 24  11.79 (4.20) |  | *n* = 24  10.79 (3.92) | *n* = 30  10.43 (4.11) |  | *N* = 108  10.71 (4.19) |
| Interest in Psychotherapy:  *M* (*SD*) | *n* = 30  14.67 (4.13) | *n* = 26  15.46 (4.26) |  | *n* = 26  16.42 (4.02) | *n* = 29  16.24 (4.36) |  | *N* = 111  15.68 (4.20) |
| IRI: |  |  |  |  |  |  |  |
| *Empathic Concern:*  *M* (*SD*) | *n* = 29  15.66 (2.35) | *n* = 26  16.65 (2.12) |  | *n* = 25  16.84 (2.59) * | *n* = 29  14.83 (3.38) * |  | *N* = 109  15.95 (2.75) |
| *Perspective Taking*  *M* (*SD*) | *n* = 29  16.24 (2.29) | *n* = 26  15.54 (2.50) |  | *n* = 25  15.72 (2.82) | *n* = 30  15.20 (2.68) |  | *N* = 110  15.67 (2.45) |
| *Fantasy*  *M* (*SD*) | *n* = 29  14.62 (2.46) | *n* = 26  14.88 (3.67) |  | *n* = 25  15.92 (2.74) | *n* = 30  15.17 (2.82) |  | *N* = 110  15.13 (2.94) |
| *Personal Distress*  *M* (*SD*) | *n* = 29  10.41 (3.04) | *n* = 26  10.65 (3.15) |  | *n* = 25  11.40 (3.16) * | *n* = 30  9.40 (2.47) * |  | *N* = 110  10.42 (3.00) |
| BIPM:  *M* (*SD*) | *n* = 29  20.26 (2.16) | *n* = 25  21.12 (2.34) |  | *n* = 24  20.29 (3.21) | *n* = 30  19.57 (2.02) |  | *N = 108*  20.27 (2.47) |

*Note.* IG: Intervention group, CG: Control group; IRI: Interpersonality Reactivity Index; BIPM: Balanced Index of Psychological Mindedness. All completed questionnaire sections were included.

** p* < .05.
